# Placental Pathology and Neuroimaging Correlates in Neonates with Congenital Heart Disease

**DOI:** 10.1038/s41598-019-40894-y

**Published:** 2019-03-11

**Authors:** Sarah D. Schlatterer, Jonathan Murnick, Marni Jacobs, Linda White, Mary T. Donofrio, Catherine Limperopoulos

**Affiliations:** 1 Division of Fetal and Transitional Medicine, Children’s National Health System, Washington, DC 20010 United States; 2 Department of Diagnostic Imaging and Radiology, Children’s National Health System, Washington, DC 20010 United States; 3 Division of Biostatistics and Study Methodology, Children’s Research Institute, Children’s National Health System, Washington, DC 20010 United States; 4 Division of Cardiology, Children’s National Health System, Washington, DC 20010 United States; 5 Department of Pediatrics, The George Washington University School of Medicine, Washington, DC 20052 United States

## Abstract

Congenital heart disease (CHD) is an independent risk factor for brain injury, including stroke, and poor neurodevelopmental outcomes, and placental abnormalities may represent an additional risk factor for brain injury in neonates. The incidence and scope of placental pathology and relationship to fetal brain abnormalities in pregnancies complicated by fetal CHD has not been explored to our knowledge. In order to determine the prevalence of placental pathology findings and whether placental findings are associated with postnatal brain injury in pregnancies complicated by fetal CHD, we reviewed placental pathology reports for 51 pregnancies complicated by CHD and scored available postnatal, pre-operative brain MRI for brain pathology. Overall, 57% of CHD infants had abnormal placental pathology. Pregnancies complicated by CHD with aortic obstruction (AO) were significantly more likely than those with no obstruction to have abnormal placental pathology (79% vs. 44%). There was a trend toward more severe brain lesions amongst patients with brain lesions and placental abnormality (55% moderate/severe) compared to those without placental abnormality (11% moderate/severe). These data suggest that placental abnormalities are common in CHD and may have a compounding effect on brain lesions in this high-risk population.

## Introduction

The incidence and scope of placental pathology and its relationship to fetal brain anomalies in pregnancies complicated by fetal congenital heart disease (CHD) is an area of study that is in its infancy. However, available evidence suggests that placentas of pregnancies complicated by CHD differ from placentas of control pregnancies. Low placental weight to birth weight ratios and additional abnormalities such as thrombosis, infarction, chorangiosis, and hypomature villi have been associated with fetal CHD^1^. Additionally, Ruiz and colleagues have shown an increase in placenta-related complications among women carrying fetuses with CHD^2^. While current studies suggest that there is no difference in placental volume in CHD vs. healthy control pregnancies, the rate of growth seems to differ^3^. A recent placental perfusion study revealed a significant difference between placental perfusion in CHD pregnancies vs. controls^4^.

In recent years, exciting evidence has emerged that the placenta may have a role in protecting the developing brain, and dysfunctional placenta may be associated with brain abnormalities. Ascending infections of the placenta and fetal thrombotic vasculopathy have been associated with neurological morbidity^5^. Histologic chorioamnionitis has been linked with encephalopathy and brain injury in other high-risk populations^6–9^. Fetal thrombotic vasculopathy has been significantly associated with perinatal brain injury, but few associations have been found between placental parenchymal infarction and fetal/neonatal brain lesions.

Given that CHD is an independent risk factor for stroke and poor neurodevelopmental outcomes and that placental abnormalities may represent a risk factor for neurologic abnormalities, we set out to explore the interplay between congenital heart disease, placental pathology, and brain lesions in this hypothesis-generating study. In the context of a prospective observational study^10,11^, we describe the prevalence and scope of placental pathology findings in a cohort of pregnancies with fetal CHD, describe the relationship of placental abnormalities to type of CHD lesion, and evaluate whether placental findings are associated with postnatal, pre-operative brain lesions in pregnancies complicated by fetal CHD.

## Results

### Placental Abnormalities and Infant Birth Characteristics

We obtained 51 placental pathology reports for pregnancies complicated by fetal congenital heart disease. Overall, 57% of CHD infants had abnormal placental pathology (Table 1). The most common primary placental abnormality was infarction (28% of infants, 48% of abnormal placental findings), followed by “other” abnormalities (16% of infants, 28% of abnormal placental findings) (Table 1). Within our cohort over 60% were male (Table 2). Mean gestational age at birth and birthweight were 38 weeks (+/−1.9 weeks) and 3069 g (+/−689.5 g), respectively. Approximately 41% of pregnancies (21/51) were of mothers 35 years old or older.

**Table 1:** Placental Pathology.

PLACENTA CHARACTERISTICS	n	%
Placental pathology Normal Abnormal	22 29	43 57
Primary placental abnormality No abnormality noted Infarction Histologic chorioamnionitis Calcifications Other	22 14 4 3 8	43 28 8 6 16

**Table 2:** Infant and Placental Characteristics and Specific Placental Abnormalities.

Infant Characteristics	Dichotomous			Primary placental abnormality				
	None	Abnormal	p-value^a^	Infarction	Chorio.	Calcific.	Other	p-value^b^
Gender (n, %) Male Female	13 (59) 9 (41)	19 (66) 10 (35)	0.77	7 (50) 7 (50)	3 (75) 1 (25)	2 (67) 1 (33)	7 (88) 1 (13)	0.50
Gestational age (median)	38.9	38.7	0.98	38.6	39.2	38.7	38.9	0.68
Birth weight (median)	3017	3108	0.22	3008	3790	2937	3104	0.11
Head circumference (median)	33.9	33.5	0.90	33.0	36.0	34.8	33.7	**0.04**
**Placental Characteristics**								
Placental weight (median)	384.5	488.0	**0.02**	443.0	656.5	452.0	484.2	**0.03**
Placental weight percentile GA (n, %) < 3^rd^ percentile ≥ 3^rd^ percentile	15 (68) 7 (32)	10 (35) 19 (66)	**0.02**	7 (50) 7 (50)	0 (0) 4 (100)	0 (0) 3 (100)	3 (38) 5 (63)	**0.02**
Placental weight percentile BW (n, %) < 3^rd^ percentile ≥ 3^rd^ percentile	16 (73) 6 (27)	11 (38) 18 (62)	**0.02**	7 (50) 7 (50)	1 (25) 3 (75)	0 (0) 3 (100)	3 (38) 5 (63)	0.06
Placenta: birth weight ratio (n, %) < 3^rd^ percentile ≥ 3^rd^ percentile	16 (73) 6 (27)	16 (55) 13 (45)	0.25	8 (57) 6 (43)	1 (25) 3 (75)	2 (67) 1 (33)	3 (38) 5 (63)	0.47

We found no significant differences in gestational age at delivery, birth weight, or gender when comparing infants of pregnancies with placental abnormalities vs. without placental abnormalities (Table 2). When groups were subdivided into cohorts based upon type of placental pathology, signficantly higher infant head circumference was noted in the group with histologic chorioamnionitis compared to no placental abnormalities (p = 0.04) and compared to pregnancies complicated by placental infarction (p = 0.03), adjusted for multiple comparisons (Table 2).

There was no significant difference in mode of delivery, elective vs. STAT c-section, induced vs spontaneous labor, APGAR scores at 1 or 5 minutes, the frequency of delivery complications, the frequency of pregnancy complications, or the need for respiratory resuscitation at birth in pregnancies with placental abnormalities compared to those with normal placental pathology reported (Table 3). Fetal distress/bradycardia was noted most frequently among delivery complications, occurring in 12% (6/51) of deliveries studied. Hypothyroidism was the most common pregnancy complication, occurring in 12% (6/51) of pregnancies. All but one pregnancy (5/6) complicated by hypothyroidism also had placental abnormalities, and the hypothyroidism group accounted for 17% (5/29) of pregnancies with placental abnormalities. While the odds ratio of having hypothyroidism in a pregnancy with placental abnormality compared with no placental abnormality was 3.89 (CI = 0.42–35.86), the finding did not reach significance, with p = 0.23.

**Table 3:** Pregnancy and Delivery Characteristics.

Pregnancy and Delivery Characteristics	Placental Findings		p-value
	None	Abnormal	
**Delivery mode** (n, %) Vaginal C-section	10 (37.0) 11 (47.8)	17 (63.0) 12 (52.2)	0.57
**C-section delivery** (n, %) Elective Stat	4 (44.4) 7 (50.0)	5 (55.6) 7 (50.0)	1.00
**Labor** (vaginal only) (n, %) Induced Spontaneous	6 (50.0) 4 (26.7)	6 (50.0) 11 (73.3)	0.26
**Pregnancy Complications** (n, %)	8 (36)	16 (41)	0.18
**Delivery Complications** (n, %**)**	5 (23)	9 (31)	0.51
**Respiratory Resuscitation** (n, %)	11 (50)	15 (52)	0.90
**APGAR 1 minute** (mean)	7.0	7.3	0.50
**APGAR 5 minute** (mean)	8.3	7.9	0.29

Pregnancy Characteristics: diabetes, pregnancy-induced hypertension, hyperthyroidism, preterm labor, placenta previa, IUGR, oligohydramnios, group-B strep positivity, anemia, morbid obesity, reduced lung capacity, and one case of transverse myelitis during pregnancy; Delivery characteristics: fetal bradycardia/distress, vacuum or forceps assist, placental abruption, nuchal cord, and hemorrhage antenatal or postnatal; Respiratory Resuscitation: requiring respiratory resuscitation beyond standard bulb suction and stimulation.

### Placental Abnormalities and Placental Characteristics

**Figure 1:**
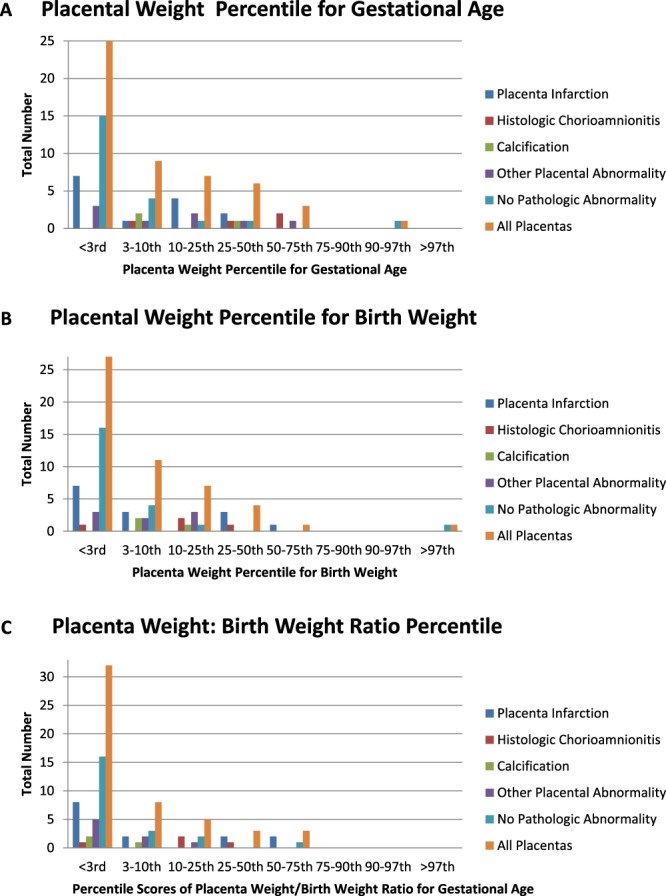
Placental Weights. (**A**) Placental weight percentiles were calculated according to normative values corrected for gestational age and gender and stratified by type of placental pathology. (**B**) Placental weight percentiles were calculated according to normative values corrected for birth weight and gender and stratified by type of placental pathology. (**C**) Placental weight: birth weight ratio was calculated and compared with normative values for gestational age and gender. Percentile ranges are reported here.

Overall, the placentas in our study were small when corrected for gestational age and birthweight (Fig. 1A,B). Placental weight (PW):birth weight (BW) ratio corrected for gestational age (GA), was also overall low in our cohort (Fig. 1C). Interestingly, placentas with pathologic abnormalities appear to be larger, both overall and based upon percentile rank from normative values (Table 2). Placentas with abnormalities overall were less likely to fall into the <3^rd^ percentile when corrected for GA and BW compared to normal placentas (35% (7/29) vs. 68% (15/22) corrected for GA and 38% (11/29) vs. 73% (16/22) corrected for BW). When the group with pathologic abnormalities was stratified into specific abnormalities, histologic chorioamnionitis placentas were found to be larger than those with no abnormalities (p = 0.07 and p = 0.04 for placental weight and GA corrected percentile, respectively, adjusted for multiple comparisons). There was no significant difference in placental weight: birth weight ratio between groups.

### Placental Abnormalities and Cerebroplacental Ratio

All patients in our cohort had at least one fetal echocardiogram for diagnostic purposes. In 50/51 cases, both umbilical artery resistive index (UA RI) and middle cerebral artery resistive index (MCA RI) were obtained on initial fetal echocardiogram. We used the UA RI and MCA RI to calculate the cerebroplacental ratio (CPR), a marker of placental function that has been associated with adverse pregnancy outcomes^12^. We found no association between placental abnormalities and abnormal CPR (defined as a CPR <1.0) on fetal echocardiogram. Among patients with an abnormal placenta, 6.9% had abnormal CPR on initial fetal echocardiogram compared to 19% of normal placentas with abnormal CPR (p = 0.22). An additional follow-up fetal echocardiogram was obtained in 25/51 cases, and, when the CPR from the second fetal echocardiogram was included in our analysis, there was still no association noted. In this combined analysis, 16% of patients with an abnormal placenta had abnormal CPR on any fetal echocardiogram compared to 19% of patients with normal placentas (p = 0.71). Patients who had a second fetal echocardiogram were more likely to have an abnormal placenta (58.6% of abnormal compared to 38.1% of normal). Of note, median gestational age at initial fetal echocardiogram was 28 weeks (+/−4.3 weeks). Median gestational age at second fetal echocardiogram amongst the 25 patients who had a second echocardiogram was 35.6 weeks (+/−2.1 weeks).

### CHD Pathology and Placental Findings

The majority of our cases had two-ventricle CHD without aortic obstruction (24/51, 47%) (Table 4). Overall, 19/51 (37%) of our cohort had single ventricle CHD and 18/51 (35%) had aortic obstruction (Table 4). Pregnancies complicated by CHD with aortic obstruction were significantly more likely than those with no obstruction to have abnormal placental pathology (83% vs. 42%, p = 0.007) (Table 5). The odds of abnormal placental pathology were 6.79 times higher among pregnancies complicated by CHD with aortic obstruction compared to those without (95% CI 1.64–28.04). The odds of having abnormal placental pathology that fell into our “other” category (i.e. dysmaturation, chronic inflammation) was 10.56 times higher among pregnancies complicated by CHD with aortic obstruction versus no obstruction (95% CI 1.61–69.12) (Table 5). No differences were seen when comparing single vs. two-ventricle CHD pathology, or additional placental pathologies.

**Table 4:** CHD Classification.

CHD Classification	n	%
**CHD Class** Class 1: 2 ventricle, no aortic obstruction - Transposition of the great arteries in intact ventricular septum (n = 8) - Transposition of the great arteries with ventricular septal defect (n = 1) - Tetralogy of Fallot (n = 9) - Tetralogy of Fallot with pulmonary atresia (n = 1) - Truncus Arteriosus (n = 1) - Total anomalous pulmonary venous connection (n = 1) - Other, 2V (n = 3) Class 2: 2 ventricle, aortic obstruction - Ventricular septal defect with interrupted aortic arch (n = 2) - Coarctation of the aorta with aortic arch hypoplasia (n = 3) - Other, 2V* (n = 3) Class 3: 1 ventricle, no obstruction - Pulmonary atresia with intact ventricular septum (n=1) - Other, 1V (n = 8) Class 4: 1 ventricle, aortic obstruction - Hypoplastic Left Heart Syndrome (n = 9) - Other, 1V* (n = 1)	24 8 9 10	47 16 18 20
**Ventricular subtype** Single Two	19 32	37 63
**Aortic obstruction subtype** No aortic obstruction Aortic obstruction	33 18	65 35

Other, 2 V = Ebstein’s anomaly (n = 1), AV canal defect (n = 2); Other 2 V* = coarctation with atrioventricular canal defect (n = 1), aortic stenosis (n = 1), biscuspid aortic valve, abnormal mitral valve, with coarct & VSD (n = 1); Other, 1 V = double outlet right ventricle (n = 3), tricuspid atresia with ventricular septal defect and pulmonary stenosis (n = 2), heterotaxy (n = 1), right ventricle dominant atrioventricular canal defect (n = 1); Other, 1 V* = DORV w/coarctation of the aorta.

**Table 5:** Placental Pathology and CHD Pathology.

CHD Pathology: Aortic Obstruction					
Placental pathology	No AO, n (%)	AO, n (%)	p-value^a^	OR	95% CI
Overall Normal Abnormal	19 (58) 14 (42)	3 (17) 15 (83)	**0.007**	**6.79**	**1.64–28.04**
Pathology None Infarction Chorioamnionitis Calcifications Other	19 (58) 8 (24) 2 (6) 1 (3) 3 (9)	3 (17) 6 (33) 2 (11) 2 (11) 5 (28)	**0.03**	4.75 6.33 12.67 **10.56**	0.95–23.84 0.63–63.64 0.86–186.89 **1.61–69.12**
**CHD Pathology: Ventricle**					
**Placental pathology**	**Single**, n (%)	**Double**, n (%)	**p-value** ^a^	**OR**	**95% CI**
Overall Normal Abnormal	9 (47) 10 (53)	13 (41) 19 (59)	0.77	1.32	0.42–4.13
Pathology None Infarction Chorioamnionitis Calcifications Other	9 (47) 4 (21) 1 (5) 2 (11) 3 (16)	13 (41) 10 (31) 3 (9) 1 (3) 5 (16)	0.81	1.73 2.08 0.35 1.15	0.41–7.29 0.19–23.30 0.03–4.42 0.22–6.10

### Preoperative Brain MRI Characteristics

Overall, 36/51 (71%) infants underwent a postnatal, pre-operative MRI per our protocol including T1, T2, diffusion-weighted imaging (DWI), susceptibility-weighted imaging (SWI), and magnetic resonance spectroscopy (MRS), allowing for complete scoring of these MRIs (Table 6). MRIs were only included if performed at <2 weeks of age as we felt any brain lesions noted after 2 weeks of age would be difficult to relate to placental findings. The majority of patients had a pre-operative MRI within the first 5 days of life.

**Table 6:** Comparison of Brain MRI Abnormalities of Patients with Normal vs. Abnormal Placental Pathology.

BRAIN MRI ABNORMALITIES	Placenta		p-value^a^
	Normal	Abnormal	
Brain MRI Abnormality Items	n (%)	n (%)	
WMI None Any	13 (77) 4 (24)	16 (76) 5 (24)	1.00
WMI None (0) Mild (1) Moderate/severe (2+)	13 (77) 3 (18) 1 (6)	16 (76) 1 (5) 4 (19)	0.29
IP hemorrhage None Any	16 (94) 1 (6)	19 (91) 2 (10)	1.00
Punctate lesions None Any	16 (94) 1 (6)	18 (86) 3 (14)	0.61
IVH None Any	15 (88) 2 (12)	18 (86) 3 (14)	1.00
SDH Normal Any	14 (82) 3 (18)	16 (76) 5 (24)	0.71
SDH Normal Minimal Spread	14 (82) 3 (18) 0 (0)	16 (76) 2 (10) 3 (14)	0.30
Brain MRI Abnormality Score	n (%)	n (%)	
Brain Lesion None Any	7 (44) 9 (56)	9 (45) 11 (55)	1.00
Brain Lesion None (0) Mild (1 - 5) Moderate/severe (6+)	7 (44) 8 (50) 1 (6)	9 (45) 5 (25) 6 (30)	0.14
Total score (median)	1.7 ^b^	1.5 ^b^	0.57

WMI: white matter injury. IP hemorrhage: intraparenchymal hemorrhage. IVH: intraventricular hemorrhage. SDH: subdural hemorrhage. Brain Injury: Complete Score denotes results of infants with complete MRI (total = 36). Of note, lactate peak, DSVT, and cerebral infarction were also scored. No patients with lactate peak or DSVT. There was a single patient with small cerebral infarction (severity score 1). ap-value based on Fisher’s exact test or Wilcoxon-Man n-Whitney test (total score). bFor reference: means = 1.7 and 3.4 (respectively).

Incomplete scans (infant unable to complete MRS in one case and unable to complete both MRS and T1 imaging in another) occured in 2 cases, allowing for partial scoring of these MRIs. In addition, a total of 13/51 cases were not scored for brain lesions in this study, either because the infant’s MRI occurred after 2 weeks of age (in 3 cases) or because pre-operative MRI was not performed, in most cases because the infant was felt to be hemodynamically unstable. One patient expired before any imaging was performed.

In total, 56% of CHD neonates (20/36) had some type of brain lesion on the MRI Abnormality Scoring System^11^. The most common type of brain lesion was white matter injury, identified in 9/38 (24%) infants. The second most common MRI finding was subdural hemorrhage, occurring in 21% (8/38). No infants in our cohort demonstrated lactate peak on MRS or deep sinus venous thrombosis (DVST). Only one patient had a small cerebral infarction (severity score 1).

### Brain Injury Outcomes and Placental Abnormalities

We compared MRI abnormality characteristics of CHD infants with normal vs. abnormal placental pathology (Tables 6 and 7). Overall, there was no statistically significant difference in brain MRI abnormality outcomes based on placental abnormalities, either overall or by type of placental pathology. However, among those with a MRI abnormalities, there was a trend towards more severe brain lesions in patients with abnormal placenta (55% moderate/severe with placental abnormality compared to 11% moderate/severe without placental abnormality) (Table 7).

**Table 7:** Odds of Brain MRI Abnormality Among Participants with Placental Abnormalities vs. No Placental Abnormalities.

	Crude		Adjusted^a^	
Brain MRI Abnormality Items	OR	95% CI	OR	95% CI
WMI	1.02	0.23–4.57	0.96	0.18–5.14
WMI severity Mild vs. none Moderate/severe vs. none	1.20 0.27 3.25	0.27–5.37 0.02–2.92 0.32–32.74	1.24^b^ 0.26 13.20	0.24–6.48 0.02–2.82 0.25–707.42
IP hemorrhage	1.68	0.14–20.33	1.90	0.15–24.72
Punctate lesions	2.67	0.25–28.28	3.30	0.28–38.85
IVH	1.25	0.18–8.49	1.38	0.19–9.89
SDH	1.46	0.29–7.23	1.44	0.28–7.34
SDH severity	1.68	0.34–8.38	1.60^b^	0.32–8.12
**Brain MRI Abnormality Score**				
Brain injury	0.95	0.25–3.57	0.93	0.24–3.58
Brain injury severity Mild vs. none Moderate/severe vs. none	1.53^b^ 0.49 4.67	0.44–5.27 0.11–2.16 0.45–48.26	1.49^b^ 0.47 9.61	0.42–5.33 0.10–2.14 0.49–188.08

Results of logistic or ordinal regression. aAdjusted for gender and gestational age at birth. bViolation of proportional odds assumption likely; polychotomous logistic regression parameters provided in addition, if possible.

We also compared MRI abnormality scores of infants delivered vaginally versus by c-section. Over 90% of infants delivered vaginally had MRI data available to calculate MRI abnormality scores, compared with less than 60% of infants delivered by c-section. We found no significant difference in brain MRI abnormality scores between these two groups. Of the 13 infants delivered by c-section that had available MRI data, infants delivered by elective c-section were more likely to have white matter injury than STAT c-section (66.7% vs. 0.0%, p = 0.02). Infants delivered by elective c-section also had higher brain MRI abnormality scores (median = 3.0 vs. 0.5, p = 0.05) than those delivered by STAT c-section. No differences were noted based on spontaneous vs. induced labor amongst infants delivered vaginally.

## Discussion

We demonstrate that placental abnormalities are common in pregnancies complicated by CHD. Over 50% of pregnancies in our cohort demonstrated a placental abnormality, with the most common feature being placental infarction, occurring in 28%. Interestingly, we found that CHD lesions with aortic obstruction were 6.79 times more likely to have placental pathology. In our cohort, brain MRI abnormalities occurred in 56% (20/36) of CHD infants overall. Among infants with brain MRI abnormalities, moderate-to-severe brain lesions occured in 55% with placental abnormality versus only 11% without placental abnormality, although our cohort was too small for these findings relating brain lesions to placental pathology to reach statistical signficance.

Recently, Rychik and colleagues reported that placental weight to birth weight ratios were abnormally low in newborns with CHD, and that placental abnormalities such as thrombosis, infarction, chorangiosis, and hypomature villi occur frequently in this population^1^. It has been proposed that differences may be evident in placental development, vascularization, oxygen, or nutrient exchange in placentas of CHD fetuses compared with controls^3^. Structural and vascular placental abnormalities have also been shown to be associated with hypoplastic left heart syndrome^13^, and a study of fetal thrombotic vasculopathy of the placenta revealed that a significant number of cases had congenital heart disease^14^. There is alteration in expression of angiogenic factors in CHD pregnancies, suggestive of impaired placental angiogenesis^15^.

In keeping with these prior studies suggesting that there is abnormal vascularization^4,13,15^ and smaller size of the placenta in CHD^1^, we found that placentas in our study were overall small, that placental efficiency was reduced compared with published normal controls^16^ of the same gestational age, gender, and birth weight, and that placental infarction was the most common abnormality (28% of pregnancies studied). Interestingly, placentas with abnormalities noted on pathology reports were significantly more likely to be above the 3^rd^ percentile in weight than placentas without any pathologic findings, although placentas overall were small (Fig. 1). We did not find any difference in placental weight: birth weight ratio when comparing abnormal to normal placental pathology. This begs the question of whether the relatively higher placental weights in the abnormal group were due to edema related to injury or whether there is some protective or adaptive mechanism underlying the small size of the placentas that is interrupted in placentas with abnormalities. Ongoing studies are needed to address this intriguing preliminary finding.

We used the cerebroplacental ratio calculated from the MCA RI and UA RI on fetal echocardiogram as a surrogate for placental function, and found no significant association between placental pathology and CPR. Interestingly, a higher percentage of infants with normal placental pathology had abnormal CPR on initial echocardiogram, although the difference was not significant. Given that CPR is a calculated value, these data may be more reflective of abnormal MCA resistive indices in the CHD population^17^ than placental function *per se*. Further studies with new tools may be required to accurately measure placental function *in vivo*. It is possible that we do not find a significant association between CPR and placental function due to the timing of the fetal echocardiogram. Initial echocardiogram was performed at a median gestational age of 28 weeks, while average gestational age at delivery was 38.9 weeks and 38.7 weeks for infants with normal and abnormal placental pathology, respectively. The percentage of patients with abnormal placenta an an abnormal CPR on any echocardiogram increased when a second echocardiogram was included in analysis, which may reflect that infants with an abnormal placenta were more likely to have a second echocardiogram.

Our data showed that pregnancies complicated by CHD with aortic obstruction were significantly more likely than those with no obstruction to have abnormal placental pathology. The association between CHD with aortic obstruction and placental abnormalities was highest for those placental abnormalities categorized as “other,” which included a heterogeneous group of diagnoses including placentas that could be classified as inflammatory lesions and dysmaturation (accelerated maturation). One prior study noted smaller placental size with CHD diagnoses of tetralogy of Fallot, double-outlet right ventricle, and major ventricular septal defects^18^, while a second study found overall low placental weight-to-birth weight ratios for newborns with CHD, similar to our study^1^.

Possible links between between CHD with aortic obstruction and placental abnormalities warrant further exploration. It has been suggested that heart and placental development are connected via common molecular signaling pathways during early development^19–23^. It is possible that early alterations in signaling pathways such as WNT, NOTCH, and Connexin 43 concomittantly lead to congenital heart disease and altered vascularization of the placenta, making the placenta less resilient to altered flow dynamics and relative hypoxia that can occur with certain types of congenital heart disease. When considering the specific case of aortic obstruction, the altered flow dynamics of fetal circulation itself could contribute to the increased placental pathology noted in our study. In the fetal circulation, the placental circulation has low resistance, and it stands to reason that altered flow dynamics resulting from obstruction could disproportionately affect placental circulation. Interestingly, in our study, placentas with abnormal pathology were overall slightly larger than placentas with no pathology, raising the question of whether those placentas possibly experienced greater alterations in blood flow, leading to congestion or edema resulting in relatively higher placental weights at birth. Ongoing prospective studies are needed to address these intriguing initial findings.

Although very few studies have examined the relationship between placental pathology and brain MRI abnormalities in newborns with CHD, numerous studies have shown an association between placental abnormalities and fetal/neonatal morbidity and mortality in other high-risk fetal-neonatal populations^24–27^. In a study of neonatal hypoxic ischemic encephalopathy (HIE), chorioamnionitis and diffuse chronic villitis correlated with severity of encephalopathy^6^. The same study showed that severity of diffuse chronic villitis was a predictor of abnormal neurodevelopmental outcome following hypothermia in infants with neonatal HIE^6^. In preterm infants born to mothers with preterm premature rupture of membranes, histologic chorioamnionitis has been associated with periventricular leukomalacia and intraventricular hemorrhage^7^. In preterm infants, histologic, but not clinical, chorioamnionitis was shown to be significantly associated with intraventricular hemorrhage, ventriculomegaly, increased periventricular echodensity, and seizures^8^. In a study in the Netherlands of hypoxic ischemic encephalopathy, chronic villitis was associated with basal ganglia and thalamic injury as well as white matter/watershed injury^9^.

Of the 51 patients with placental pathology reports in our study, postnatal, pre-operative MRI was completed within 2 weeks of birth in over 70%. In our relatively limited sample, we did not find a statistically significant correlation between placental pathology abnormalities and brain MRI abnormalitiesabnormalities. It is known that CHD confers an independent increased risk of brain injury, and, because of this, a larger sample size than available in this study will likely be required in order to make any firm associations between placental pathology and brain MRI abnormalities in CHD. Although our findings did not reach statistical significance, there was a trend noted amongst newborns with brain injury towards more severe brain injury in cases with placental abnormalities. This trend and evidence in the literature suggesting that placental function may impact brain injury and neurodevelopmental outcomes justifies further investigation into the connection between placenta, congenital heart disease, and brain injury in future studies with larger sample sizes and prospective design.

Our data do not suggest an association between abnormal placental pathology and markers of a difficult transition (i.e. lower APGAR scores, increased need for respiratory resuscitation at birth). There was no significant difference between groups with no placental pathology versus placental pathology with regards to infant birth weight, gestational age at delivery, pregnancy complications, delivery complications, 1- or 5-minute APGAR scores, need for respiratory resuscitation at delivery, or mode of delivery. It is possible that the brain injury noted in these newborns was more closely associated with early postnatal hemodynamic instability and circulation than with transition from prenatal to postnatal life or intrauterine environment.

Interestingly, the majority of cases missing MRIs in our cohort were delivered by c-section. Over 40% of infants delivered by c-section did not undergo a preoperative MRI, while less than 10% of infants delivered vaginally did not have a preoperative brain MRI study. Amongst the small number of infants delivered by c-section that had available MRI data (n = 13), infants delivered by elective c-section were more likely to have white matter injury than infants delivered by STAT c-sections. Infants delivered by elective c-section also had higher injury scores than infants delivered by STAT c-section. While we do not have records regarding clinical reasoning for mode of delivery for these infants due to all infants being born outside of our stand-alone children’s hospital, we hypothesize that these findings are likely more related to the underlying reason for elective vs. STAT c-section. Fetuses with more severe CHD are more likely to be delivered by elective c-section, and white matter injury may be more related to type of CHD. It is likely that a STAT c-section was performed in response to fetal or maternal stress or decompensation during labor, and may have prevented brain injury by preventing prolonged labor in these cases. The number of infants delivered by c-section that also had available MRI data are small, and it is difficult to draw any meaningful conclusion for this reason. Future studies are needed to address these intriguing preliminary findings.

Given our relatively small sample size, we chose to include subjects with pregnancy complications in our study. We recognize that including patients with additional pregnancy complications may skew results in a study directed at linking the cause of placental pathology with CHD, but that was not the purpose of this study. Hypothyroidism was fairly common in our cohort (6/51, 12%), and all but one pregnancy with hypothyroidism also had placental abnormalities. To our knowledge, associations between hypothyrodisim and placental pathology have not been explored. However, one study of pregnant women in the Netherlands showed that increasing levels of placental angiogenic factors were associated with decreasing levels of maternal thyroid hormone, suggesting that placental development may affect maternal thyroid hormone^28^. Although we were insufficiently powered to perform robust statistical analyses, these data do suggest that there may be interplay between the placenta and thyroid function. Maternal hypothyroidism and placental pathology and function may be an interesting area for further study.

We excluded any infant with a known genetic or chromosomal abnormality, extracardiac abnormalities suggesting a genetic syndrome or chromosome abnormality, congenital infection, or multiple gestation from this study in an effort to prevent confounding from these factors. It is known that approximately 20% of CHD cases are associated with genetic or chromosomal abnormalities^29,30^. A recent prospective study found that chromosomal abnormalities were detected by microarray analysis in approximately 50% of infants with CHD plus additional structural abnormalities or CHD plus IUGR^30^. In our center, genetic testing is typically offered for all patients with congenital heart disease, but not all families choose to pursue this option. Conotruncal abnormalities, including tetralogy of Fallot are significantly associated with 22q11 deletion. In adults, 22q11 deletion syndrome has been associated with brain MRI abnormalities such as T2 white matter bright foci, midline anomalies, cerebral atrophy or ventricular enlargement, and mild cerebellar atrophy^31^. Additionally, tetralogy of Fallot has been associated with mutations in a variety of genes, including gata4, nkx2.5, jag1, foxc2, tbx5, and tbx1^32^. TBX1 is involved in brain vascularization in mouse models^33^. Septal defects are frequently associated with trisomy 21, which has been associated with overall smaller volumes of both gray and white matter in the brain^34^. While we have excluded any cases with known genetic or chromosomal abnormalities in this study, it is possible that undiagnosed genetic abnormalities exist in our study population, and genetic defects may confound interpretation of brain MRI abnormalities.

The limitations of our study also deserve mention. The placental pathology results in this study are based upon placental pathology reports from birth hospitals within our catchment area, because we were not able to obtain the tissue for independent review. Because placental tissue was not available to us due to the retrospective nature of our design, we used the diagnostic report made by the pathologist at each site. It is likely that each pathologist had his/her own reporting style. Future prospective studies are needed to address this key point. Our overall sample size was relatively small and the type of CHD diagnostic groups were heterogeneous, limiting our ability to examine the relationship between brain MRI abnormalities, placental pathology, and specific types of CHD. For this reason, we have chosen, for the most part, to group our cohorts broadly (i.e. single vs. two ventricle pathology; no aortic obstruction vs. aortic obstruction and abnormal vs. normal placental pathology) for most analyses. The overall prevalence of placental pathology in the larger CHD cohort may be under- or overestimated by this study, given that the placenta was sent for pathology in only 56% of our newborns with CHD. It is unclear from available data why some placentas were sent for pathology while others were not. Given the small size of this study, it was not possible to control for CHD type. With these limitations in mind, we consider this pilot study to be a hypothesis-generating one that will be the basis for future, prospective studies in this field.

To our knowledge, this is the first study to examine the relationship between postnatal preoperative brain MRI abnormalities, placental pathology, and CHD. Future, prospective studies in a larger sample including an independent review of placental pathology will help further elucidate the link between CHD subtype, specific placental pathologies, and brain injury. Our data reveal a link between aortic obstruction and placental pathology that deserves further study. While we did not find a statistically significant effect of placental pathology on brain injury, we did find a trend towards more severe brain lesionslesionsamong newborns with brain MRI abnormalities with placental pathology, suggesting placental function may have subtle or compounding effects in brain injury in CHD. Such effects may only be elucidated by a larger sample size and may be related to the type of CHD. Overall, these data suggest that further investigation into the placenta-heart-brain connection is warranted.

## Methods

In the context of a single-center, prospective, observational study^4,35^ we recruited pregnant women whose fetus was diagnosed with CHD. Infants with documented genetic or chromosomal abnormalities, congenital infection, or multiple gestation were excluded from the study. All infants in our study had CHD diagnoses confirmed prenatally with fetal echocardiogram. Patients were recruited from the Fetal Heart Program Clinic at the Children’s National Health System. The Institutional Review Board of Children’s National Health System approved this study, and written informed consent was obtained from each participant. This study was performed in accordance with the institutional regulations and guidelines.

### Placental Pathology

Placental pathology reports from the birth hospitals of 51 infants with CHD were obtained for patients enrolled in our study. Placental characteristics, including placental weight, umbilical arteries/veins, and intact vs. ill-formed cotelydons were noted. Reports were reviewed for evidence of any placental structural abnormality. We used normative values for gestational age and gender for singleton pregnancies reporded by Almog and colleagues^16^ to determine placental weight percentiles. Placentas were grouped into the following cohorts: no pathologic findings (normal) vs. abnormal placenta. The abnormal placenta group was further subdivided into placental infarction, histologic chorioamnionitis, placental calcifications, and other placental abnormality. Abnormalities classified as “other” are as follows: chronic deciduitis, plasmacytic, patchy villlous agglutination and focal villitis; eccentric cord with disrupted area at cord insertion; markedly disrupted placenta with variable maturation with edema of villi and few foci of chronic lymphohistiocytic villitis, patchy chorioangiosis; small chorioangiomahemangioma; microscopic focus of avascular vili, single hyalinized decidual vessel with thrombosis and fibrinoid necrosis; accelerated villous maturation and focal villous edema; subchorionic fibrin depositis on <5% of fetal surface; villous hypermaturation with small villi.

While calcifications are thought to be a marker for placental maturity^10^, they may also be a marker of placental dysfunction. All newborns in our study were delivered at 40 weeks or earlier, and those newborns noted to have abnormal placental calcifications were 39 weeks or less. Calcifications were counted as abnormal in 3 cases: one with calcifications covering >40% of the placental surface, one with microcalcifications and a cyst on the fetal surface of the placenta, and one with noted dystrophic calcifications.

Histologic chorioamnionitis classification was based upon the placental pathology report. We did not have data regarding maternal fever and other markers of clinical chorioamnionitis available. Notation of chorioamnionitis, even mild, on the placental pathology report was regarded as abnormal in this study. Any placental infarction was considered abnormal, regardless of size or location.

### Cerebroplacental Ratios

All patients in our cohort were diagnosed with congenital heart disease prentally by fetal echocardiogram. In 50/51 cases, at least one fetal echocardiogram measured MCA RI and UA RI. We calculated the cerebroplacental ratio (CPR) as CPR = MCA RI/UA RI^36^. We considered CPR <1.0 as abnormal and CPR ≥1.00 as normal for the purposes of our analysis^36,37^. We used Fisher’s exact test to determine whether a significant correlation exists between CPR and placental pathology findings.

### Medical Record Abstraction

We reviewed the medical records of the infants and mother-fetal dyads in the study, including CHD diagnosis, prenatal maternal conditions, pregnancy and delivery complications, APGAR scores, birth weight, head circumference, and gestational age at birth of the infant, and need for resuscitation at birth.

### CHD Diagnostic Categories

Congenital heart disease diagnoses were classified into 4 groups (or classes) by our cardiologist (MTD) based upon the type of lesion noted on fetal echocardiogram. Class 1 included CHD with 2 ventricles and no obstruction to aortic flow. Class 2 included CHD with two ventricles and associated aortic obstruction. Class 3 included CHD with one functioning ventricle and no aortic obstruction. Class 4 included CHD with one functioning ventricle and aortic obstruction. From this classification, comparisons were made between CHD with single vs. two ventricle physiology and CHD with aortic obstruction vs. no obstruction.

### Conventional MRI Studies

Imaging was performed on a 1.5 T or 3 T magnet and included conventional T1, T2, DTI, and GRE sequences, as well as MR spectroscopy of the basal ganglia. Postnatal, pre-operative brain MRI was reviewed by our neuroradiologist (JM) and scored according to the Magnetic Resonance Imaging Abnormality Scoring System used by Androupoulos *et al*.^11^. Brain MRI abnormalities were given a score of 0–3, with 0 = none, 1 = mild, 2 = moderate, 3 = severe in the following subcategories: white matter injury (WMI), infarction (INF), intraparenchymal hemorrhage (IPH), punctate lesions (PL), lactate peak on MRS (lactate), intraventricular hemorrahge (IVH), subdural hemorrahge (SDH), or deep venous sinus thrombosis (DVST). Each subcategory score was multiplied by a significance multiplier factor of 1–3, as follows. WMI, INF, and IPH had a significance multiplier of 3. PL and lactate had a significance multiplier of 2. IVH, SDH, and DVST had a significance multipler of 1. Total scores were calculated by adding scores from all subcategories after each score was multiplied by its significance multiplier.

### Statistical analysis

We used Fisher’s exact test or Wilcoxon-Mann-Whitney to calculate p-values when comparing characteristics of infants, placenta, and brain MRI abnormality scores when placenta was grouped into normal vs. abnormal placenta. We used Fisher’s exact test or Krushkal-Wallis to compare characteristics of infants, placenta, and brain injury when across placental pathology categories; the Dwass-Steel-Critchlow-Fligner test was used for adjusted pairwise comparisons between categories for continuous varaibles. Logistic regression, both crude and adjusted for gender gestational age at birth, was used to estimate odds for dichotomous outcomes. Ordinal regression was used for categorical severity scores, with odds ratios from polychotomous logistic regression presented, when possible, to account for violations of the proportional hazards assumption. A two-tailed significance level of p < 0.05 was used for all tests.

## Data Availability

The datasets generated during and/or analysed during the current study are available from the corresponding author on reasonable request.

## References

[1] Rychik, J. *et al*. Characterization of the Placenta in the Newborn with Congenital Heart Disease: Distinctions Based on Type of Cardiac Malformation. *Pediatr Cardiol*, 10.1007/s00246-018-1876-x (2018). 10.1007/s00246-018-1876-x PMC6096845 29728721

[2] Ruiz A (2016). Placenta-related complications in women carrying a foetus with congenital heart disease. J Matern Fetal Neonatal Med.

[3] Andescavage N (2015). 3-D volumetric MRI evaluation of the placenta in fetuses with complex congenital heart disease. Placenta.

[4] Zun Z, Limperopoulos C (2018). Placental perfusion imaging using velocity-selective arterial spin labeling. Magn Reson Med.

[5] Roescher AM, Timmer A, Erwich JJ, Bos AF (2014). Placental pathology, perinatal death, neonatal outcome, and neurological development: a systematic review. PLoS One.

[6] Mir IN (2015). Placental pathology is associated with severity of neonatal encephalopathy and adverse developmental outcomes following hypothermia. Am J Obstet Gynecol.

[7] Lu HY, Zhang Q, Wang QX, Lu JY (2016). Contribution of Histologic Chorioamnionitis and Fetal Inflammatory Response Syndrome to Increased Risk of Brain Injury in Infants With Preterm Premature Rupture of Membranes. Pediatr Neurol.

[8] De Felice C (2001). Early neonatal brain injury in histologic chorioamnionitis. J Pediatr.

[9] Harteman JC (2013). Placental pathology in full-term infants with hypoxic-ischemic neonatal encephalopathy and association with magnetic resonance imaging pattern of brain injury. J Pediatr.

[10] Grannum PA, Berkowitz RL, Hobbins JC (1979). The ultrasonic changes in the maturing placenta and their relation to fetal pulmonic maturity. Am J Obstet Gynecol.

[11] Andropoulos, D. B. *et al*. Brain immaturity is associated with brain injury before and after neonatal cardiac surgery with high-flow bypass and cerebral oxygenation monitoring. *J Thorac Cardiovasc Surg* **139**, 543–556, doi:S0022-5223(09)01076-9 (2010). 10.1016/j.jtcvs.2009.08.022 PMC2827639 19909994

[12] Khalil A (2017). Is cerebroplacental ratio a marker of impaired fetal growth velocity and adverse pregnancy outcome?. Am J Obstet Gynecol.

[13] Jones HN (2015). Hypoplastic left heart syndrome is associated with structural and vascular placental abnormalities and leptin dysregulation. Placenta.

[14] Saleemuddin A (2010). Obstetric and perinatal complications in placentas with fetal thrombotic vasculopathy. Pediatr Dev Pathol.

[15] Llurba E (2014). Maternal and foetal angiogenic imbalance in congenital heart defects. Eur Heart J.

[16] Almog B (2011). Placenta weight percentile curves for singleton and twins deliveries. Placenta.

[17] Itsukaichi M (2011). Changes in fetal circulation associated with congenital heart disease and their effects on fetal growth. Fetal Diagn Ther.

[18] Matthiesen NB (2016). Congenital Heart Defects and Indices of Placental and Fetal Growth in a Nationwide Study of 924 422 Liveborn Infants. Circulation.

[19] Courtney JA, Cnota JF, Jones HN (2018). The Role of Abnormal Placentation in Congenital Heart Disease; Cause, Correlate, or Consequence?. Front Physiol.

[20] De Falco M (2007). Expression and distribution of notch protein members in human placenta throughout pregnancy. Placenta.

[21] de la Pompa JL, Epstein JA (2012). Coordinating tissue interactions: Notch signaling in cardiac development and disease. Dev Cell.

[22] Dor Y (2001). A novel role for VEGF in endocardial cushion formation and its potential contribution to congenital heart defects. Development.

[23] Dunk CE (2012). The molecular role of connexin 43 in human trophoblast cell fusion. Biol Reprod.

[24] Gibbins KJ (2016). Stillbirth, hypertensive disorders of pregnancy, and placental pathology. Placenta.

[25] Bukowski R (2017). Altered fetal growth, placental abnormalities, and stillbirth. PLoS One.

[26] Zeng J, Marcus A, Buhtoiarova T, Mittal K (2017). Distribution and potential significance of intravillous and intrafibrinous particulate microcalcification. Placenta.

[27] Nigam J (2014). Histopathological study of placentae in low birth weight babies in India. Ann Med Health Sci Res.

[28] Korevaar TI (2015). Placental Angiogenic Factors Are Associated With Maternal Thyroid Function and Modify hCG-Mediated FT4 Stimulation. J Clin Endocrinol Metab.

[29] Blue GM, Kirk EP, Sholler GF, Harvey RP, Winlaw DS (2012). Congenital heart disease: current knowledge about causes and inheritance. Med J Aust.

[30] Wang Y (2018). Prenatal chromosomal microarray analysis in fetuses with congenital heart disease: a prospective cohort study. Am J Obstet Gynecol.

[31] Chow EW (1999). Qualitative MRI findings in adults with 22q11 deletion syndrome and schizophrenia. Biol Psychiatry.

[32] Morgenthau A, Frishman WH (2018). Genetic Origins of Tetralogy of Fallot. Cardiol Rev.

[33] Cioffi S (2014). Tbx1 regulates brain vascularization. Hum Mol Genet.

[34] Pinter JD, Eliez S, Schmitt JE, Capone GT, Reiss AL (2001). Neuroanatomy of Down’s syndrome: a high-resolution MRI study. Am J Psychiatry.

[35] De Asis-Cruz J, Donofrio MT, Vezina G, Limperopoulos C (2018). Aberrant brain functional connectivity in newborns with congenital heart disease before cardiac surgery. NeuroImage. Clinical.

[36] Arias F (1994). Accuracy of the middle-cerebral-to-umbilical-artery resistance index ratio in the prediction of neonatal outcome in patients at high risk for fetal and neonatal complications. Am J Obstet Gynecol.

[37] DeVore GR (2015). The importance of the cerebroplacental ratio in the evaluation of fetal well-being in SGA and AGA fetuses. Am J Obstet Gynecol.

